# Glycosyl benzoates as novel substrates for glycosynthases†

**DOI:** 10.1039/d3ob00979c

**Published:** 2023-07-19

**Authors:** Sabrina de Lorenzo, Lauriane Pillet, David Lim, Francesca Paradisi

**Affiliations:** a Department of Chemistry, Biochemistry, and Pharmaceutical Sciences, University of Bern Freiestrasse 3 CH-3012 Bern Switzerland francesca.paradisi@unibe.ch david.lim@unibe.ch

## Abstract

The development of a procedure for the one-pot synthesis of glycosyl benzoates directly from unprotected sugars in aqueous media using 2-chloro-1,3-dimethylimidazolium chloride (DMC), thiobenzoic acid, and triethylamine is reported. These glycosyl donors are excellent substrates for wild-type and mutant glycosidases. β-Glucosyl benzoate was hydrolysed by the GH1 β-glucosidase derived from *Halothermothrix orenii* (*Hor*GH1). Subsequent use of this substrate in thioligase-mediated glycosylation of *p*-nitrothiophenol demonstrated their superiority as donors compared to their *p*-nitrophenol counterparts with excellent conversions. Using a series of arene nucleophiles, we also demonstrate good to excellent conversions (up to 94%) of β-glucosyl benzoate to the corresponding *p*-nitrophenyl- and thioglycosides.

## Abstract 2

An engineered glycosidase from *H. orenii* showcases for the first time that glycosyl benzoates are better donors than p-nitrophenyl glucoside in enzyme-catalysed thioglucosylations. The thioglycoside products were formed with conversions up to 94%.

## Graphical abstract

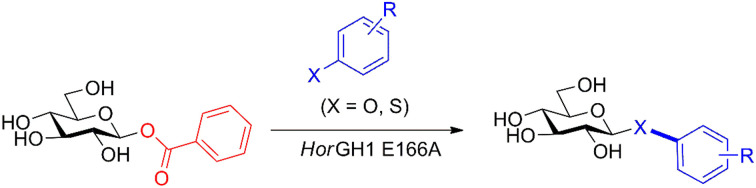

## Introduction

Mimetics of carbohydrates that are resistant towards enzymatic hydrolysis have found value as therapeutics. For example, truncated variants of the natural MUC1 mucin are found overexpressed in many tumor tissues. Accordingly, when short epitopes bearing different tumor-associated carbohydrate antigens, such as the Tn determinant (α-*O*-GalNAc-Ser/Thr), are presented to the immune system, these molecules are recognized by anti-MUC1 antibodies.^1^ However, due to these structures possessing the natural O-glycosidic linkage, these molecules are susceptible to degradation by endogenous glycosidases, leading to shorter half-lives. To overcome this issue and similar drawbacks, strategies to incorporate unnatural linkages into these molecules has been implemented.^2^ Compañón *et al.* demonstrated that an O → S/Se replacement at the glycosidic linkage led to an increase in the carbohydrate-peptide distance and observed a change in both the dynamics and orientation of the glycosidic linkage.^3^ Consequently, the new glycopeptides demonstrated improved binding towards a representative anti-MUC1 antibody. A major drawback in the synthesis of these important molecules lies in the fact that complex protecting group strategies are required which are technically demanding and generate a lot of waste. Therefore, there has been a drive to develop methods for the synthesis of glycomimetics possessing similar unnatural linkages.

Although stereoselective glycosylation has made significant progress within the past century, the chemical synthesis of glycoconjugates often still requires the use of protecting group strategies to introduce the desired linkages.^4^ Alternatively, biocatalytic methods are becoming increasingly attractive due to their excellent regio/stereoselectivity and their high efficiency while requiring much milder conditions.^5,6^ In particular, the use of mutant glycosidases, aptly termed glycosynthases, has allowed the stereoselective formation of glycosidic linkages. However, the use of glycosynthases necessitates the supplementation of activated glycosyl donors to compensate for the inability of the mutant to activate the glycosidic bond. Several classes of glycosyl donors have been reported for glycosynthases, including 4-nitrophenyl glycosides^7^ and glycosyl azides,^8^ fluorides,^9^ or oxazolines.^10^ The use of glycosyl acetates as substrates for glycosidases has been reported, albeit seldomly.^11,12^ Moreover, their use as substrates for glycosynthases has never, to-date, been reported. This may stem from the fact that this donor lacks a suitable chromophore for UV analyses of reaction mixtures.

Glycosyl esters have high potential as substrates for glycosidases and glycosynthases. For example, Zinin *et al.* demonstrated that the value of *k*_cat_ for the hydrolysis of 1-*O*-acetyl-β-d-galactopyranose by β-galactosidase from *Penicillium* sp. was three orders of magnitude greater than for *p*-nitrophenyl β-d-galactopyranoside.^11^ This finding is hardly surprising due to the higher p*K*_a_ of *p*-nitrophenol (7.15) compared to acetic acid (4.76), rendering the latter a better leaving group. These facts prompted us to investigate the possibility of glycosyl esters also being better substrates for glycosynthases.

Herein, we report the synthesis of glycosyl benzoates using DMC. These substrates were then screened against glycosyl hydrolases and glycosynthases. The improved leaving group ability of benzoate was demonstrated by comparing the glycosylation of this substrate against common donors, such as *p*-nitrophenyl glycosides, thereby making glycosyl benzoates better sugar donors than their counterparts.

## Results and discussion

### Synthesis of glycosyl benzoates using DMC

**Scheme 1:**
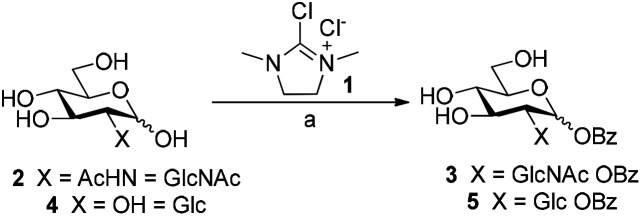
Direct synthesis of glycosyl benzoates from unprotected sugars using DMC. Reaction conditions: 2 or 4, thiobenzoic acid (5 equiv.), Et_3_N (10 equiv.), D_2_O/CD_3_CN (5 : 1), 0 °C, then 1 (3 equiv.), 30 min. 3: 22%; 5: 17%.

We initially investigated the formation of glycosyl benzoates using the dehydrative reagent, 2-chloro-1,3-dimethylimidazolium chloride 1 (DMC).^13^ Prior reports suggested that free sugars could be transformed into the corresponding glycosyl acetate using thioacetic acid and DMC, affording the corresponding products in high yields and stereoselectivity.^12^ We envisaged that the use of commercially available thiobenzoic acid should yield a similar outcome. However, initial screens were quick to show that the decreased solubility of thiobenzoic acid in water necessitated the use of co-solvent in the reaction mixtures. In this vein, we treated *N*-acetyl-d-glucosamine 2 with thiobenzoic acid and DMC in a range of co-solvent mixtures (H_2_O/MeCN, H_2_O/dioxane, H_2_O/THF) and ratios (Scheme 1, Table S1, ESI†).

The use of MeCN as co-solvent, in a ratio of 5 : 1, gave the best yield of 22% and an anomeric mixture α : β of 4 : 1 (Table S1, entry 1, ESI†). We then moved to convert d-glucose 4 to the corresponding benzoate 5 using the same conditions (Table S2, ESI†). Similarly, we observed that the use of MeCN as the co-solvent, either as a ratio of 5 : 1 or 2 : 1, afforded 5 in a yield of 17%, exclusively as the β anomer. Further attempts to optimise the conversion by variation of reaction temperature, increasing reagents equivalents, iterative addition of reagents,^12^ or modifying the co-solvent ratios did not lead to any significant improvements (Fig. S1 and Tables S3, S4, ESI†). With our optimised procedure in hand, we subjected a series of unprotected mono-, di-, and trisaccharides to the reaction conditions. Subsequent isolation of the products by preparative RP-HPLC gave the corresponding glycosyl benzoates 7–10 in similar low yields (Fig. S5, ESI, entries 2–4, 3–11% yield†). While the galactosyl benzoate did form under our reaction conditions, we were unable to separate galactosyl benzoate 6 from formed by-products (Table S5, ESI, entry 1†). The rationale for the stereoselective formation of esters for sugars bearing a 2-OH was recently elucidated by Qiu *et al.*^14^ whereby any formed 1,2-*cis*-glycosyl acetates undergo migration of the ester from the anomeric position to 2-OH and, accordingly, we also isolated migration products by RP-HPLC. Moreover, the low yields observed in our DMC-mediated esterification, compared to that of the earlier work is likely due to the insolubility of thiobenzoic acid under our reaction conditions.

### Glycosyl benzoates as substrates for glycosyl hydrolases

**Scheme 2:**
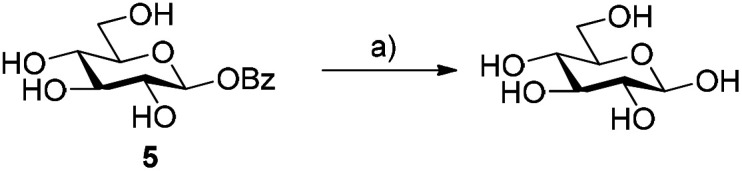
Use of β-glucosyl benzoate as a substrate for β-glucosidase from *H. orenii*. Reagents and conditions: (a) 1 mM β-Bz-Glc 5, HorGH1 (5.13 U), 50 mM HEPES buffer, 150 mM NaCl, pH 7.4, 25 °C, 74% after 1 h. Conversions were determined by monitoring the formation of benzoic acid over time by RP-HPLC.

We first probed the use of glycosyl benzoates to act as substrates for glycosyl hydrolases (Scheme 2). Earlier work demonstrated that β-glucosyl acetate was successfully hydrolysed and transglycosylated with MeOH using β-glucosidase from almonds.^12^ When we incubated the same substrate with β-glucosidase from *Halothermothrix orenii* (*Hor*GH1, EC 3.2.1.21), we observed rapid hydrolysis of 5 of 74% after 1 h. These values also consider the auto-hydrolysis of 5, (2% after 1 h), which reached a maximum value of 8% after 24 h. With these promising results in hand, we moved on to using 5 as a substrate for glycosynthases.

### β-Glucosyl benzoate as a substrate for *Hor*GH1 mutants

The ability of glycosyl benzoates to act as substrates for glycosynthases was then investigated. Recently, Pillet *et al.* demonstrated that several mutants of *Hor*GH1 accepted aromatic thiols as substrates for thioglycoside synthesis.^15^ In their work, the classic *p*-nitrophenyl glycoside and glycosyl fluoride were used as sugar donors for the thioglycosynthase. We surmised that glycosyl benzoates would be better substrates for these mutants due to the fact that while glycosyl fluorides are good donors for glycosynthases (HF p*K*_a_ = 3.8), these substrates lack a suitable chromophore for UV-spectroscopic monitoring, while *p*-nitrophenyl glycosides (*p*NP p*K*_a_ = 7.15) have a poorer leaving group ability compared to that of a carboxylic acid (p*K*_a_ = ∼5). In this vein, we incubated 5 with *Hor*GH1 E166A and E166A/M299R mutants in the presence of *p*-nitrothiophenol as the acceptor. Pleasingly, we observed rapid formation of β-pNT-Glc 11 reaching a maximum conversion of 94% and 92% with *Hor*GH1 E166A and *Hor*GH1 E166A/M299R mutants, respectively, after 4 h (Table 1, entries 1 and 2; Fig. S3, ESI†). Comparatively, and in accordance with the results previously obtained by Pillet *et al.*, when *p*-nitrophenyl-β-d-glucopyranoside 12 was incubated with the same enzymes under the same conditions, a low 21% and 15% conversion was observed with *Hor*GH1 E166A and *Hor*GH1 E166A/M299R mutants, respectively, after 24 h (Table 1, entries 3 and 4; Fig. S3, ESI†). These results confirm the superior leaving group ability of benzoate over *p*NP. Incubation of 5 with the acid/base and nucleophilic mutant (*Hor*GH1 E166A/E354G) gave 11 solely as the β-anomer (Table 1, entry 5). Given that *Hor*GH1 E166A/E354G has lost both its acid/base and nucleophilic residues, we would expect that an activated β-glycosyl donor should not be accepted as a substrate. The observation that we see the β-linked product 12 suggests that the enzyme catalyses the reaction following an S_N_1-type pathway, whereby the scaffold of *Hor*GH1 E166A/E354G promotes a ping-pong like mechanism, assisting the departure of the benzoyl group, followed by subsequent thioglycosylation. Interestingly, incubation of 5 with the triple mutant (*Hor*GH1 E166A/E354G/M299R) yielded no thioglycoside 11 (Table 1, entry 6).

**Table 1:** Synthesis of *p*-nitrothiophenyl-β-d-glucopyranoside 11 using HorGH1 mutants

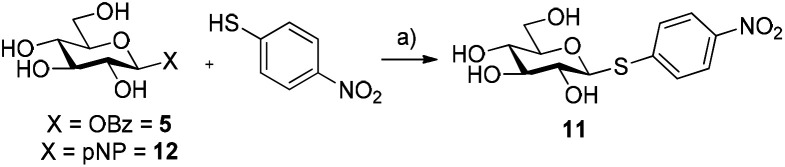			
Entry	Substrate	Mutant	Conversion [%]
1	β-Bz-Glc 5	E166A	94
2	β-Bz-Glc 5	E166A/M299R	92
3	β-pNP-Glc 12	E166A	21
4	β-pNP-Glc 12	E166A/M299R	15
5	β-Bz-Glc 5	E166A/E354G	8^a^
6	β-Bz-Glc 5	E166A/E354G/M299R	0

a Only β-linked thioglycoside was observed.

Encouraged by these findings, we then decided to screen the scope of nucleophiles capable of being accepted by the acid/base mutants under these conditions. The use of alternative aryl thiols 13a–c as acceptors yielded the corresponding thioglycosides in good to excellent conversions (72–89%, Table 2, entries 1–3; Fig. S4, ESI†), while no conversion was observed with *p*-aminothiophenol 13d or *N*,*N*′-dibenzoylcysteine 13e (Table 2, entries 4 and 5, respectively). Interestingly, amongst the aryl alcohols, *p*NP 13f gave the corresponding β-pNP-Glc 12 in 45% conversion after 1 h (Table 2, entry 6). We found that after this time, 12 gradually hydrolyses to d-glucose under the reaction conditions, reaching 22% of 12 after 24 h. This result suggests that 12 is a substrate for *Hor*GH1 E166A and is hydrolysed by the enzyme over time. This is further supported by the fact that 12, in the absence of enzyme, was not observed to hydrolyse under the reaction conditions (data not shown). Moreover, doubling the concentration of *p*NP (10 mM to 20 mM) led to a slower rate of hydrolysis of the corresponding product (38% after 24 h, Fig. S5, ESI†). Interestingly, the reaction of 5 with 13f gave the corresponding product with β-stereochemistry. The more electron-rich *p*-methoxyphenol 13g and phenol 13h did not yield their corresponding products (Table 2, entries 7 and 8).

**Table 2:** Synthesis of glycosides using HorGH1 E166A

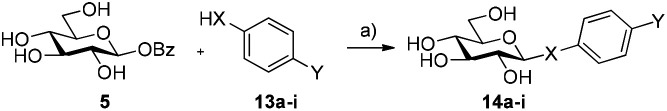		
Entry	Acceptor	Conversion [%]
1	*p*-Thiotoluene (X = S; Y = CH_3_), 13a	72
2	*p*-Bromothiophenol (X = S; Y = Br) 13b	89
3	2-Naphthalenethiol, 13c	78
4	*p*-Aminothiophenol (X = S; Y = NH_2_), 13d	0
5	*N*,*N*′-Dibenzoyl-l-cystine, 13e	0
6	*p*-Nitrophenol (X = O; Y = NO_2_), 13f	45
7	*p*-Methoxyphenol (X = O, Y = OCH_3_), 13g	0
8	Phenol (X = O, Y = H), 13h	0
9	Diphenyl diselenide, 13i	0

## Conclusions

In conclusion, we have developed a protecting-group-free synthesis of glycosyl benzoates using DMC and thiobenzoic acid. Albeit being low yielding reactions due to unwanted migratory and degradative processes, these substrates were shown to be suitable substrates for glycosidases and glycosynthases. We demonstrated that glycosyl benzoates perform much better than classic *p*-nitrophenyl donors in the thioglycosylation reaction using our single acid/base mutant *Hor*GH1 E166A and our double mutant E166A/M299R. Therefore, we provide critical insight on how to overcome the leaving group ability limitation in the thioglycoligase strategy for the enzymatic synthesis of thioglycosides. Moreover, by mitigating the use of protecting groups, our strategy complies with the 8th principle of Green Chemistry.^16^ By expanding the repertoire of sugar donors available for glycosynthase-catalysed glycoside formation, we pave the way to more efficient synthetic routes for the selective and sustainable synthesis of these stable glycosidic analogues of glycosides present in many commercial products and pharmaceuticals.^17,18^

## Author contributions

F. P. supervised the project. D. L. conceptualized the idea, wrote the initial draft, and guided the synthesis of the glycosyl benzoates as well as the biotransformations. S.d.L. performed most of the experimental work. L. P. expressed and purified the enzyme variants and helped in the design of biotransformations. All authors discussed and agreed to the final version of the manuscript.

## Conflicts of interest

There are no conflict of interest to declare.

## Supplementary Material

OB-021-D3OB00979C-s001

## References

[cit1] Ju T., Otto V. I., Cummings R. D. (2011). Angew. Chem., Int. Ed..

[cit2] Wu X., McFall-Boegeman H., Rashidijahanabad Z., Liu K., Pett C., Yu J., Schorlemer M., Ramadan S., Behren S., Westerlind U., Huang X. (2021). Org. Biomol. Chem..

[cit3] Compañón I., Guerreiro A., Mangini V., Castro-Lopez J., Escudero-Casao M., Avenoza A., Busto J. H., Castillon S., Jimenez-Barbero J., Asensio J. L., Jimenez-Oses G., Boutureira O., Peregrina J. M., Hurtado-Guerrero R., Fiammengo R., Bernardes G. J. L., Corzana F. (2019). J. Am. Chem. Soc..

[cit4] Wan L. Q., Zhang X., Zou Y., Shi R., Cao J. G., Xu S. Y., Deng L. F., Zhou L., Gong Y., Shu X., Lee G. Y., Ren H., Dai L., Qi S., Houk K. N., Niu D. (2021). J. Am. Chem. Soc..

[cit5] Miller D. C., Athavale S. V., Arnold F. H. (2022). Nat. Synth..

[cit6] Rocha R. A., Speight R. E., Scott C. (2022). Org. Process Res. Dev..

[cit7] Meszaros Z., Nekvasilova P., Bojarova P., Kren V., Slamova K. (2021). Biotechnol. Adv..

[cit8] Cobucci-Ponzano B., Conte F., Bedini E., Corsaro M. M., Parrilli M., Sulzenbacher G., Lipski A., Dal Piaz F., Lepore L., Rossi M., Moracci M. (2009). Chem. Biol..

[cit9] Williams S. J., Withers S. G. (2000). Carbohydr. Res..

[cit10] Heidecke C. D., Parsons T. B., Fairbanks A. J. (2009). Carbohydr. Res..

[cit11] Zinin A. I., Eneyskaya E. V., Shabalin K. A., Kulminskaya A. A., Shishlyannikov S. M., Neustroev K. N. (2002). Carbohydr. Res..

[cit12] Lim D., Fairbanks A. J. (2017). Chem. Sci..

[cit13] Isobe T., Ishikawa T. (1999). J. Org. Chem..

[cit14] Qiu X., Chong D., Fairbanks A. J. (2023). Org. Lett..

[cit15] Pillet L., Lim D., Almulhim N., Benitez-Mateos A. I., Paradisi F. (2022). Chem. Commun..

[cit16] Anastas P., Eghbali N. (2010). Chem. Soc. Rev..

[cit17] Pachamuthu K., Schmidt R. R. (2006). Chem. Rev..

[cit18] Qiao M., Zhang L., Jiao R., Zhang S., Li B., Zhang X. (2021). Tetrahedron.

