# Supplementary material for: Glycosyl benzoates as novel substrates for glycosynthases
Source: Org Biomol Chem. 2023 Jul 19;21(31):6356–9. doi: 10.1039/d3ob00979c (PMC10410497; doi:10.1039/d3ob00979c)
Supplement: OB-021-D3OB00979C-s001 [file OB-021-D3OB00979C-s001.pdf]

Electronic Supplementary Material (ESI) for Organic & Biomolecular Chemistry.
This journal is © The Royal Society of Chemistry 2023

Supporting Information

Glycosyl Benzoates as Efficient Substrates for Glycosynthases

Sabrina de Lorenzo,[1] Lauriane Pillet,[1] David Lim,[1] and Francesca Paradisi[1]

[1] Department of Chemistry, Biochemistry, and Pharmaceutical Sciences, Freiestrasse 3, 3012, Bern, Switzerland

Table of Contents

Materials and Methods 3

Expression and Purification of *Hor*GH1 wild-type and mutants 4

Synthesis of Glycosyl Benzoates 4

Table S1 Conversion of GlcNAc **2** to GlcNAc **3** benzoate under different co-solvents and co-solvent ratios...4

Table S2 Conversion of Glc **4** to Glc benzoate **5** under different co-solvents and ratios......5

Figure S1 Conversion of Glc **4** to GlcOBz **5** over time...5

Table S3 Screening of Et_3N, thiobenzoic acid, and DMC equivalents in the conversion of Glc **4** to GlcOBz **5**. 6

Table S4 Iterative addition of reagents for the conversion of Glc **4** to GlcOBz **5**................6

Table S5 Selective benzoylation of the anomeric hydroxyl group of mono-, di-, and trisaccharides. 6

Screening of glycosyl esters as substrates for glycosyl hydrolases 7

Figure S2 Use of ß-glucosyl benzoate **5** as a substrate for the hydrolytic reaction by ß-glucosidases. 7

Figure S3 Formation of pNT-Glc **11** using HorGH1 E166A or E166A/M299R and using either ß-glucosyl benzoate **5** or ß-pNP-Glc **12** as the glycosyl donor...................................7

Screening of thiols accepted by *Hor*GH1 E166A mutant 8

Figure S4 Conversion of ß-Bz-Glc **5** to thioglycoside products by HorGH1 E166A.. 8

Figure S5 Use of *p*-nitrophenol as the glycosyl acceptor in HorGH1 E166A catalysed glycosylation of ß-Bz-Glc **5**. 9

Experimental 10

2-Chloro-1,3-dimethylimidazolinium chloride **1**[5] 10

Benzoyl 2-Acetamido-2-deoxy-D-glucopyranoside **3** 10

Benzoyl ß-D-glucopyranoside **5** 11

Benzoyl α-D-mannopyranoside **7** 11

Benzoyl ß-D-lactoside **8** 12

Benzoyl ß-D-maltoside **9** 13

Benzoyl ß-D-maltotrioside **10**..13

Spectral Data 15

Benzoyl 2-Acetamido-2-deoxy-D-glucopyranoside **3** 15

Benzoyl ß-D-glucopyranoside **5** 16

Benzoyl α-D-mannopyranoside **7** 17
Benzoyl ß-D-lactoside **8** 18
Benzoyl ß-D-maltoside **9** 19
Benzoyl ß-D-maltotrioside **10** 20
References 21

Materials and Methods

All commercially available chemicals were used without any further purification. Double-distilled water was retrieved from a Milli-Q® IQ 7000 Ultrapure Water System. To measure the pH of solutions, an LLG-pH Meter 7 was used. RP-HPLC analyses were conducted with an HPLC Dionex UltiMate 3000 (Thermo Fisher, Loughborough, UK) equipped with a C18 column 3.5 µm, 2.1 × 100 mm (Waters, Elstree, UK). All chemical reagents were acquired from Sigma Aldrich (Merck KGaA, Darmstadt) unless otherwise specified. 4-Nitrophenyl-β-D-glucopyranoside (*p*NP-Glc) used for activity assays was purchased from Carbosynth. All commercial chemicals were used without further purification. Standards of *p*-Nitrophenyl-β-D-thioglucopyranoside(*p*NP-Glc), *p*-toluyl-β-D-glucopyranoside and *p*-bromophenyl-β-D-thioglucopyranoside were synthetised according to literature procedures.[1] Reactions conducted at 0 °C were cooled by means of an ice bath. Reactions at -20 °C were achieved by mixing NaCl and ice (*w/w*, 1:3). Dry solvents were obtained by filtration over columns of dried alumina under argon (SPS). Solvent was removed under reduced pressure using a Buchi™ rotary evaporator. Thin Layer Chromatography (t.l.c.) was carried out on Merck Silica Gel 60F254 aluminium-backed plates. Visualisation of the plates was achieved using a UV lamp (λ_{max} = 254 or 365 nm), and/or ammonium molybdate (5% in 2 M H_2SO_4), and/or aniline-diphenylamine-85 % phosphoric acid (4 mL : 4 g : 20 mL) in acetone (96 mL) and/or ninhydrin. Flash column chromatography was carried out using Silicycle SiliaFlash® P60 silica (230-400 mesh). Proton and carbon nuclear magnetic resonance (δ_H, δ_C) spectra were recorded on a Bruker Ascend™ 300 (300 MHz) spectrometer. All chemical shifts are quoted on the δ-scale in ppm using the residual solvent as an internal standard. ^{1}H and ^{13}C spectra were assigned using COSY, DEPT, HSQC, and HMBC. For mannopyranosides, a coupled HSQC spectrum was obtained in order to determine the anomeric configuration based on the $^1J_{C-H}$ value (value of a β-anomer is ~ 160 Hz; α-anomer is ~ 170 Hz).[2] High resolution mass spectra were

recorded a Bruker FT-ICR mass spectrometer using electrospray ionisation (ESI). *M/z* values are reported in Daltons.

Expression and Purification of *Hor*GH1 wild-type and mutants

DNA preparation and site-directed mutagenesis of the *Halothermothrix orenii* (*Hor*GH1, EC 3.2.1.21) mutants (E166A, E166A/M299R, E166A/E354G, E166A/E354G/M99R) was previously performed by Pillet *et al.*[1] and the same plasmids were used in this study. The wild type and mutant *Hor*GH1 variants expression, purification and quantification was performed according to Pillet *et al.*[1] Briefly, the plasmids harbouring the genes of the different variants were transformed into chemically competent *E.coli* BL21(DE3) and expression was carried out in 300 mL autoinduction media with 100 µg/mL ampicillin at 37 °C and 150 rpm for 24 hours. Cells were then centrifuged at 2500·*g*, 4 °C, 20 min, sonicated at 40 % amplitude for 8 min, with pulses of 5 s ON, 10 s OFF and purified by metal affinity chromatography (IMAC) using an AKTA™ Start. Purification of the enzyme was checked by analysing the different fractions with a 12 % SDS PAGE and the concentration of the purified enzymes was estimated by measuring the absorbance at 280 nm in the EPOCH2 (nanodrop Take3 plate) using an extinction coefficient (ε) of 106,230 $L \cdot mol^{-1} \cdot cm^{-1}$ (predicted using the ExPASy ProtParam tool[3-4]) and a molecular weight of 52,003.56 Da.[3-4]

Synthesis of Glycosyl Benzoates

Table S1 Conversion of GlcNAc **2** to GlcNAc **3** benzoate under different co-solvents and co-solvent ratios.

Entry	Co-solvent	D_2O/Co-solvent	Conversion [%]	α/ß
1	MeCN	5 : 1	22	1 : 4
2	MeCN	2 : 1	20	3 : 2
3	MeCN	1 : 1	-	-
4	Dioxane	5 : 1	16	3 : 2
5	Dioxane	2 : 1	16	3 : 2
6	Dioxane	1 : 1	1	*n.q.*
7	THF	5 : 1	7	3 : 2
8	THF	2 : 1	7	3 : 1

9	THF	1 : 1	7	3 : 1

Conversions were determined by ^{1}H NMR integration of the anomeric protons. n.q. not quantified.

Table S2 Conversion of Glc **4** to Glc benzoate **5** under different co-solvents and ratios.

Entry	Co-solvent	D_2O/Co-solvent	Conversion [%]	α/ß
1	MeCN	5 : 1	17	0 : 1
2	MeCN	2 : 1	17	0 : 1
3	MeCN	1 : 1	15	0 : 1
4	MeCN	1:2	-	-
5	Dioxane	5 : 1	12	0 : 1
6	Dioxane	2 : 1	12	0 : 1
7	Dioxane	1 : 1	12	0 : 1
8	THF	5 : 1	10	0 : 1
9	THF	2 : 1	10	0 : 1
10	THF	1 : 1	10	0 : 1

Conversions were determined by ^{1}H NMR integration of the anomeric protons. n.q. not quantified.

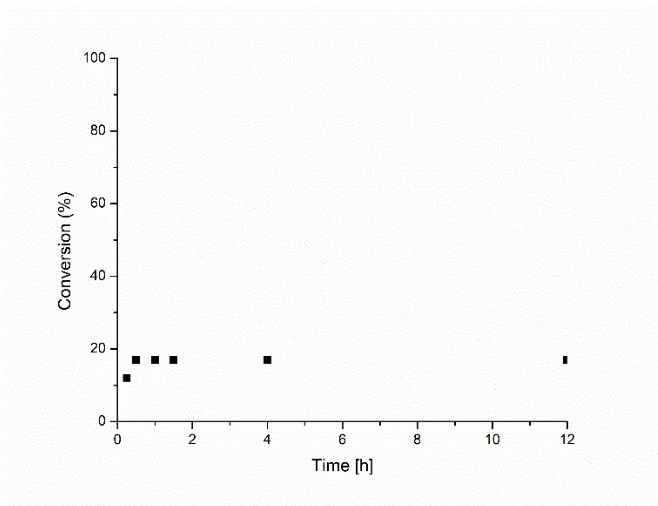

Figure S1 Conversion of Glc **4** to GlcOBz **5** over time.

Table S3 Screening of Et_3N, thiobenzoic acid, and DMC equivalents in the conversion of Glc **4** to GlcOBz **5**.

Entry	Et_3N (equiv.)	Thiobenzoic acid (equiv)	DMC 1 (equiv.)	Conversion [%]	α/ß
1	20	5	3	-	-
2	10	10	3	8	0 : 1
3	10	5	6	20	0 : 1
4	10	5	9	10	0 : 1
5	2.1	1	3	8	0 : 1
6	5	5	6	12	0 : 1

Table S4 Iterative addition of reagents for the conversion of Glc **4** to GlcOBz **5**.

Entry	Et_3N (equiv.)	Thiobenzoic acid (equiv)	DMC 1 (equiv.)	Conversion [%]	α/ß
1	10	5	2	15	0 : 1
2	10	5	3	17	0 : 1

Table S5 Selective benzoylation of the anomeric hydroxyl group of mono-, di-, and trisaccharides.

Entry	Sugar	Product	Yield [%]	α/ß
1	galactose	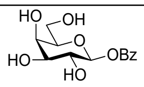 **6**	n.d.	-
2	Mannose	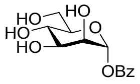 **7**	4	α only
3	lactose	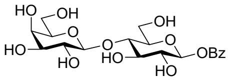 **8**	11	ß only
4	maltose	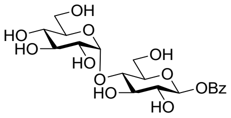 **9**	5	ß only
5	maltotriose	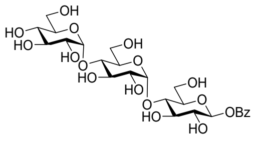 **10**	3	ß only

^{a} Reagents and conditions: Sugar, thiobenzoic acid (5 equiv.), Et_3N (10 equiv.), H_2O/MeCN (5:1), 0 °C, then DMC (10 equiv.) 30 mins.

Screening of glycosyl esters as substrates for glycosyl hydrolases

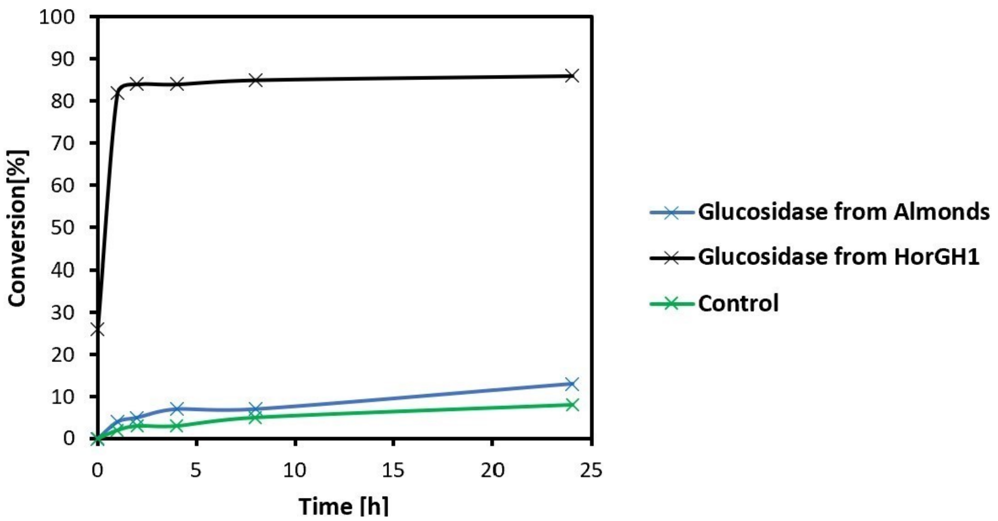

Figure S2 Use of ß-glucosyl benzoate **5** as a substrate for the hydrolytic reaction by ß-glucosidases.

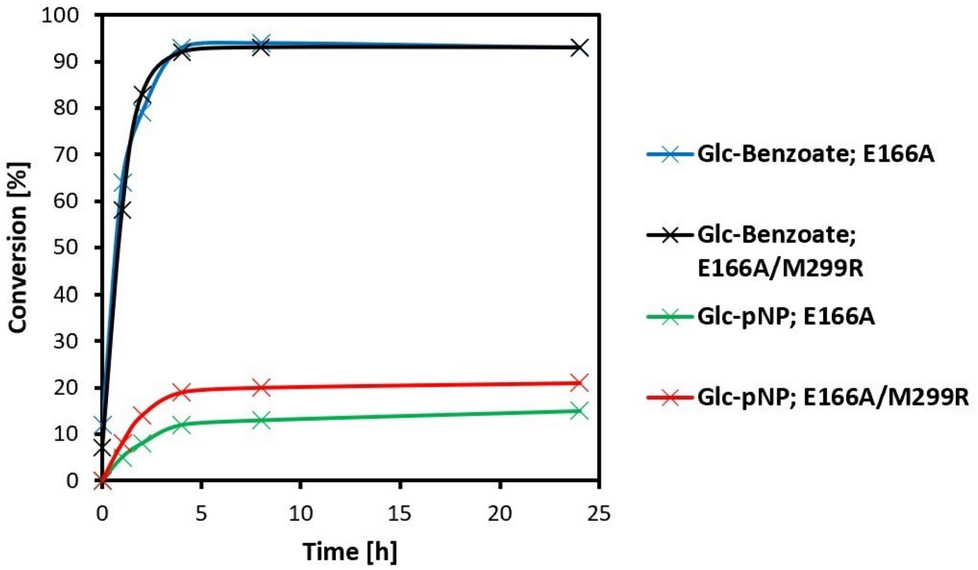

Figure S3 Formation of pNT-Glc **11** using HorGH1 E166A or E166A/M299R and using either ß-glucosyl benzoate **5** or ß-pNP-Glc **12** as the glycosyl donor.

Screening of thiols accepted by *Hor*GH1 E166A mutant

Reactions were performed with ß-D-glucopyranosyl benzoate **5** as the sugar donor and the corresponding thiols **13a-i** as the acceptor. Reactions were performed at 25 °C in 50 mM HEPES buffer pH 7.4 containing 30 % DMSO, 0.3 mg/mL enzyme, 1 mM sugar donor, 20 mM ß-mercaptoethanol and 10 mM of thiol in a total reaction volume of 1 mL.

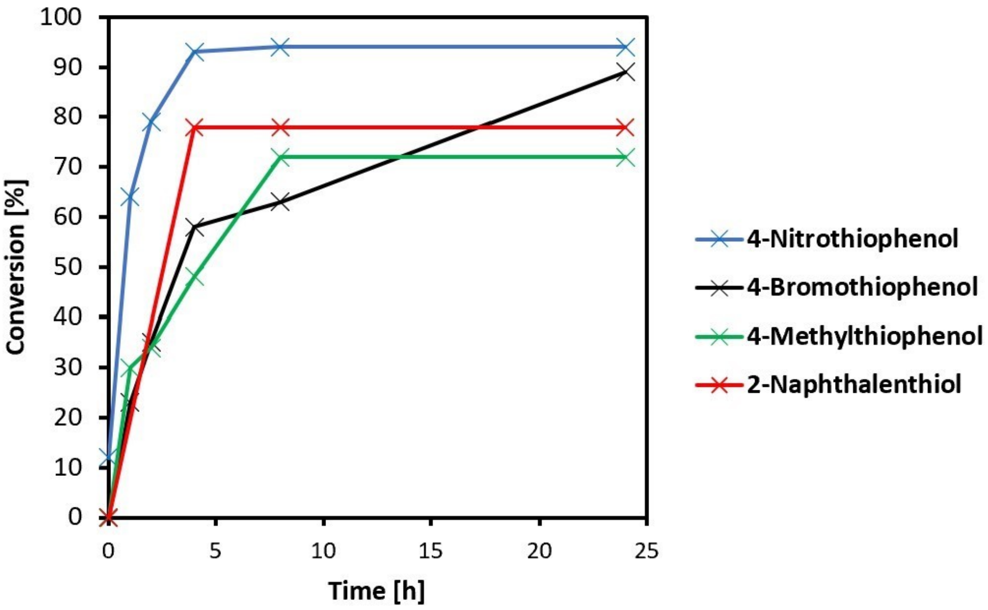

Figure S4 Conversion of ß-Bz-Glc **5** to thioglycoside products by HorGH1 E166A.

Reactions were performed at 25 °C in 50 mM HEPES buffer pH 7.4 containing 30 % DMSO, 0.3 mg/mL enzyme, 1 mM sugar donor, 20 mM ß-mercaptoethanol and 10 mM of thiol in a total reaction volume of 1 mL.

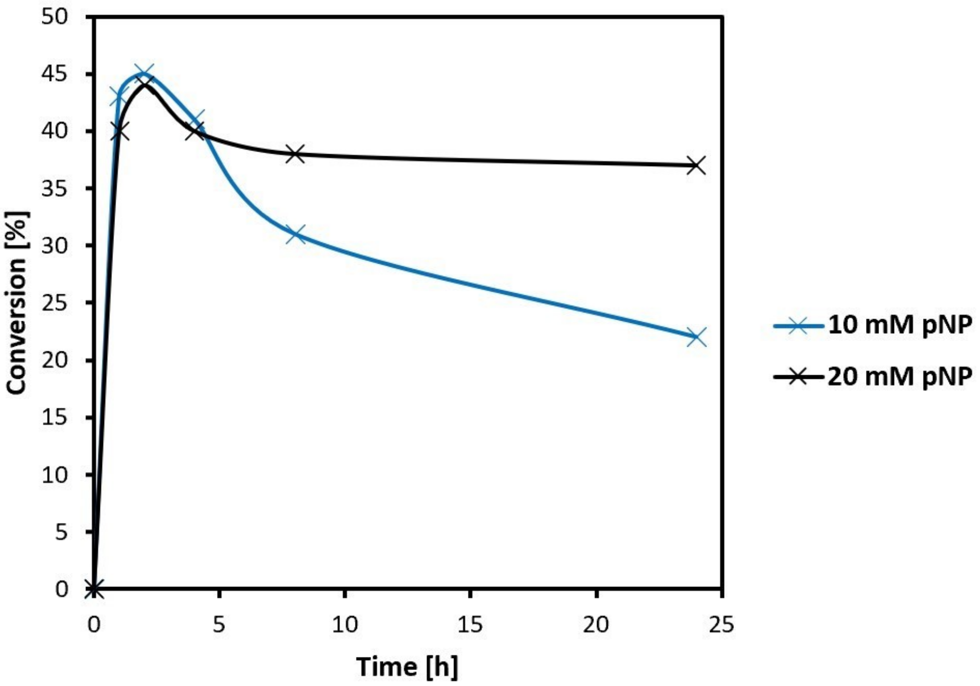

Figure S5 Use of *p*-nitrophenol as the glycosyl acceptor in HorGH1 E166A catalysed glycosylation of ß-Bz-Glc **5**.

Confirmation of β-thioglycosides synthesis by ^{1}H NMR

The stereochemistry of the thioglycosides were confirmed by ^{1}H NMR by comparing the authentic standard (Figure S6A)[1] to the enzymatic reaction performed by *Hor*GH1 E166A variant as described in the "Screening of thiols accepted by the *Hor*GH1 E166A mutant" section (Figure S6C). A control was run in parallel with all components except the enzyme (Figure S6B).

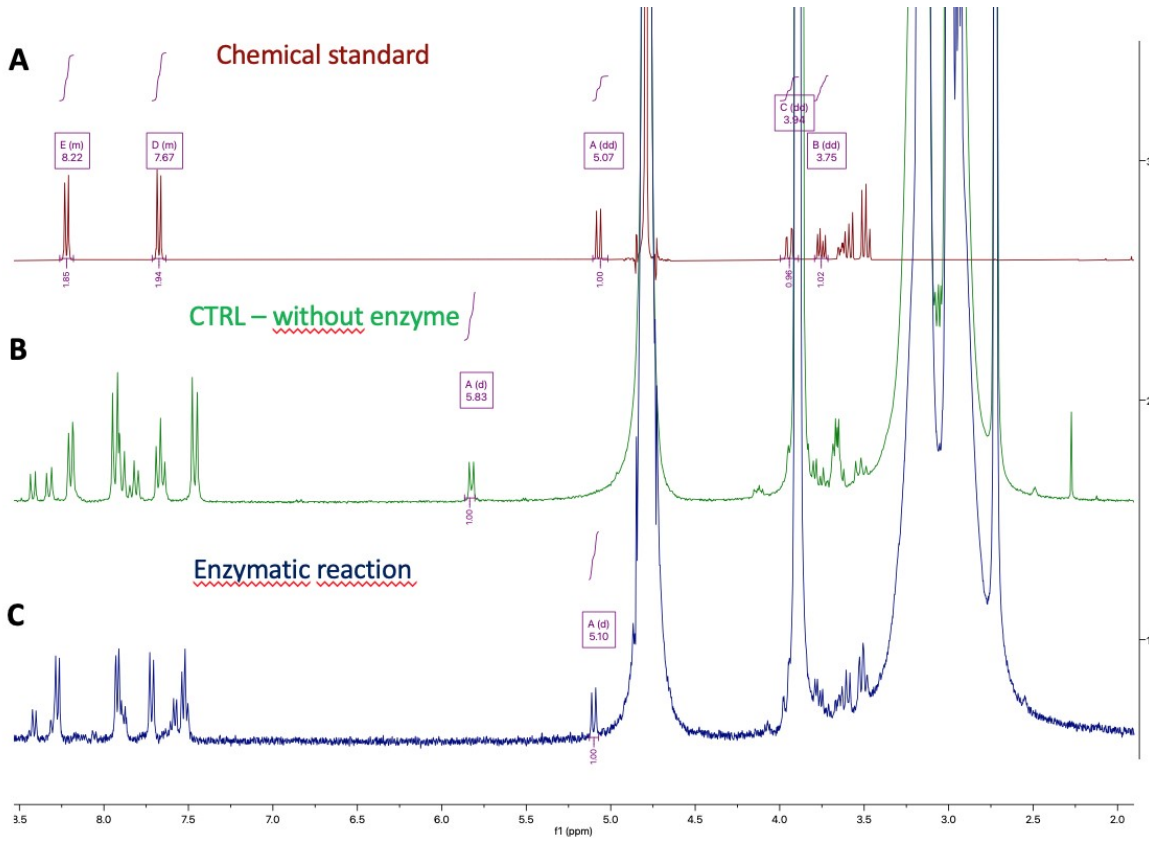

Figure S6: Overlaid ^{1}H NMR spectra of authentic *p*-nitrophenyl-β-D-thioglucopyranoside (*p*NT-Glc) (**A**), the biocatalytic reaction mixture using the *Hor*GH1 E166A mutant (**C**), and the control reaction without enzyme (**B**). Chemical shifts for the anomeric and aromatic protons of the product are provided: δ_H (300 MHz, D_2O) 5.10 (1H, d, $J_{1,2}$ 9.6 Hz, H-1), 7.71 (1H, d, *J* 9.0 Hz, Ar-H), 8.22 (1H, d, *J* 9.0 Hz, Ar-H).

Experimental

2-Chloro-1,3-dimethylimidazolinium chloride **1**[5]

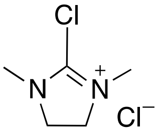

1,3-Dimethylimidazolidinone (19.0 mL, 176 mmol) was dissolved in toluene (200 mL) and stirred under an N_2 atmosphere. Oxalyl chloride (20 mL, 233 mmol) was added dropwise and the reaction was stirred at 50 °C. After 7 h, the flask was allowed to cool to rt and stirred overnight. After 15 h, the precipitate was filtered, washed with cold toluene and recrystallized (acetonitrile/diethyl ether). The white solid was filtered and dried under vacuum to yield 2-chloro-1,3-dimethylimidazolidinium chloride **1** (18.1 g, 61 %) as a white powdery solid; δ_H (300 MHz, CD_3CN)[5] 3.14 (6H, s, 2 x CH_3), 3.96 (4H, s, 2 x CH_2); HRMS (ESI) Calcd. For $C_5H_{10}ClN_2$ (M^+) 133.0527. Found 133.0529.

Benzoyl 2-Acetamido-2-deoxy-D-glucopyranoside **3**

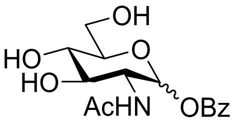

N-Acetyl-D-glucosamine **2** (120 mg, 0.55 mmol), thiobenzoic acid (320 µL, 2.75 mmol) and triethylamine (770 µL, 1.70 mmol) were stirred in H_2O/MeCN (5:1, 2 mL), and the mixture was cooled to 0 °C. DMC **1** (290 mg, 1.70 mmol) was then added portionwise and the mixture then allowed to stir for 30 mins. The reaction mixture was then diluted with H_2O (5 mL), washed with DCM (5 x 10 mL), and lyophilised. Purification of the residue by flash column chromatography gave benzoyl 2-acetamido-2-deoxy-D-glucopyranoside **3** (17.6 mg, 10 %) as a white solid (α/ß, 4:1); R_f (α anomer): 0.5 (15:85 MeOH:DCM), R_f (ß anomer): 0.4 (15:85 DCM:MeOH); δ_H (400 MHz, CD_3CN) α-anomer: 1.96 (3H, s, NHCOC$\underline{H}_3$), 3.61-3.68 (1H, m, H-5), 3.76-3.86 (3H, m, H-4, H-6, H-6'), 4.00 (1H, dd, $J_{2,3}$ 10.8Hz, $J_{3,4}$ 8.9 Hz, H-3), 4.12 (1H, dd, $J_{1,2}$ 3.5 Hz, H-2), 6.33 (1H, d, H-1), 7.59 (1H, d, J 7.2 Hz, Ar-H), 7.75 (1H, t, J 8.2 Hz, Ar-H), 8.14 (2H, d, J 7.4 Hz, Ar-H); δ_C (100 MHz, CD_3CN) 21.1 (q, NHCO$\underline{C}H_3$), 52.3 (d, C-2), 59.6 (t, C-6), 68.7 (d, C-5), 69.9 (d, C-3), 73.8 (d, C-4), 90.9 (d, C-1), 127.7, 128.3, 129.3, 133.8 (Ar-C), 165.7 (s, NH$\underline{C}$OCH_3), 173.4 (s, $\underline{C}OC_6H_5$); δ_H (400 MHz, CD_3CN) ß-anomer:

1.94 (1H, s, NHCOCH_3), 3.54 (1H, at, *J* 9.8 Hz, H-5), 3.68-3.75 (1H, m, H-6), 3.76-3.86 (2H, m, H-4, H-6'), 3.90 (1H, dd, $J_{2,3}$ 10.5 Hz, $J_{3,4}$ 8.9 Hz, H-3), 4.06 (1H, dd, $J_{1,2}$ 8.7 Hz, H-2), 5.78 (1H, d, H-1), 7.59 (2H, d, *J* 7.2 Hz, Ar-H), 7.75 (1H, d, *J* 8.2 Hz, Ar-H), 8.06 (1H, d, *J* 8.1 Hz, Ar-H); δ_C (100 MHz, CD_3CN) 21.5 (q, NHCO$\underline{C}H_3$), 53.8 (d, C-2), 60.0 (t, C-6), 69.0 (d, C-5), 72.7 (d, C-3), 76.3 (d, C-4), 93.0 (d, C-1), 128.1, 128.4, 129.3, 133.9 (Ar-C), 165.7 (s, NH$\underline{C}OCH_3$), 173.3 (s, $\underline{C}OC_6H_5$); HRMS (ESI) Cald. for $C_{15}H_{20}NO_7^+$ (M + H^+) 326.1234. Found 326.1238.

Benzoyl ß-D-glucopyranoside **5**

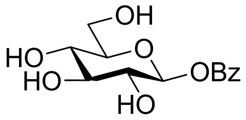

D-Glucose monohydrate **4** (2.00 g, 10.1 mmol), thiobenzoic acid (6.0 mL, 50.9 mmol) and triethylamine (14 mL, 100 mmol) were stirred in H_2O/MeCN (5:1, 36 mL), and the mixture was cooled to 0 °C. DMC **1** (5.21 g, 30.7 mmol) was then added portionwise and the mixture then allowed to stir for 30 mins. The reaction mixture was then washed with DCM (5 x 50 mL), and the aqueous phase was filtered through a 0.45 µm PTFE filter. Purification of the filtrate by semi-preparative RP-HPLC (column: Agilent 5 Prep-C18 (100 Å); eluent: A (water + 0.1 % trifluoroacetic acid) and B (MeCN + 0.1 % trifluoroacetic acid); sample was run at 25 mL/min with a gradient of: 0-30 min: 100% A, 30-40 min 100% B, 40-60 min: 100 % A, 60-70 min: 100% A; detection: 235 nm) afforded benzoyl ß-D-glucopyranoside 5 (258 mg, 9 %) as a white solid, R_f 0.4 (15:85 MeOH:DCM); t_R = 4.68 mins; δ_H (400 MHz, D_2O)[6] 3.45 (1H, m, H-4), 3.56-3.64 (3H, m, H-2, H-3, H-5), 3.68 (1H, dd, $J_{6,6'}$ 12.4 Hz, $J_{5,6}$ 5.5 Hz, H-6), 3.84 (1H, dd, $J_{5,6'}$ 2.2 Hz, H-6'), 5.75 (1H, d, $J_{1,2}$ 7.5 Hz, H-1), 7.50 (1H, at, *J* 7.8 Hz, Ar-H), 7.66 (1H, d, *J* 7.7 Hz, Ar-H), 8.05 (1H, d, *J* 7.7 Hz, Ar-H); HRMS (ESI) Cald. for $C_{13}H_{16}O_7Na^+$ (M + Na^+) 307.0788. Found 307.0797.

Benzoyl α-D-mannopyranoside **7**

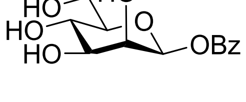

D-Mannose (200 mg, 1.12 mmol), thiobenzoic acid (670 µL, 5.60 mmol) and triethylamine (1.6 mL, 11.4 mmol) were stirred in H_2O/MeCN (5:1, 4 mL), and the mixture was cooled to 0

°C. DMC **1** (584 g, 3.40 mmol) was then added portionwise and the mixture then allowed to stir for 30 mins. The reaction mixture was then diluted with H_2O (20 mL), washed with DCM (5 x 25 mL), and the aqueous phase was filtered through a 0.45 µm PTFE filter. Purification of the filtrate by semi-preparative RP-HPLC (column: Agilent 5 Prep-C18 (100 Å); eluent: A (water + 0.1 % trifluoroacetic acid) and B (MeCN + 0.1 % trifluoroacetic acid); sample was run at 25 mL/min with a gradient of: 0-30 min: 100% A, 30-40 min 100% B, 40-60 min: 100 % A, 60-70 min: 100% A; detection: 235 nm) afforded benzoyl ß-D-mannopyranoside **7** (13.8 mg, 4 %) as a white solid, R_f 0.4 (15:85 MeOH:DCM); t_R = 4.67 mins; δ_H (400 MHz, D_2O)[6] 3.78-3.89 (4H, m, H-2, H-3, H-6, H-6'), 4.10 (1H, m, H-4), 4.18 (1H, m, H-5), 6.27 (1H, d, $J_{1,2}$ 1.9 Hz, H-1), 7.58 (2H, at, J 7.8 Hz, Ar-H), 7.76 (1H, at, J 7.5 Hz, Ar-H), 8.11 (2H, d, J 7.7 Hz, Ar-H); HRMS (ESI) Cald. for $C_{13}H_{15}O_7^-$ (M -z H^+) 283.0823. Found 283.0817.

Benzoyl ß-D-lactoside **8**

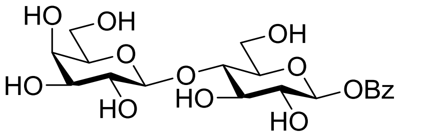

α-Lactose monohydrate (402 mg, 1.11 mmol), thiobenzoic acid (670 µL, 5.60 mmol) and triethylamine (1.60 mL, 11.4 mmol) were stirred in H_2O/MeCN (5:1, 4 mL), and the mixture was cooled to 0 °C. DMC **1** (580 g, 3.40 mmol) was then added portionwise and the mixture then allowed to stir for 30 mins. The reaction mixture was then diluted with H_2O (20 mL), washed with DCM (5 x 25 mL), and the aqueous phase was filtered through a 0.45 µm PTFE filter. Purification of the filtrate by semi-preparative RP-HPLC (column: Agilent 5 Prep-C18 (100 Å); eluent: A (water + 0.1 % trifluoroacetic acid) and B (MeCN + 0.1 % trifluoroacetic acid); sample was run at 25 mL/min with a gradient of: 0-30 min: 100% A, 30-40 min 100% B, 40-60 min: 100 % A, 60-70 min: 100% A; detection: 235 nm) afforded benzoyl ß-D-lactoside **8** (54.2 mg, 11 %) as a white solid, R_f 0.2 (1:4 MeOH:DCM); t_R = 4.82 mins; δ_H (400 MHz, D_2O)[6] 3.66 (1H, dd, $J_{2b,3b}$ 9.9 Hz, $J_{1b,2b}$ 7.7 Hz, H-2b) 3.76 (1H, dd, $J_{3b,4b}$ 3.4 Hz, H-3b), 3.81-3.95 (8H, m, H-2a, H-3a, H-4a, H-5a, H-6a, H-6a', H-6b, H-6b'), 4.02 (1H, m, H-5b), 4.06 (1H, m, H-4b), 4.57 (1H, d, $J_{1b,2b}$ 7.7 Hz, H-1b), 5.93 (1H, d, $J_{1a,2a}$ 7.9 Hz, H-1a), 7.66 (2H, at, J 7.7 Hz, Ar-H), 7.83 (1H, t, J 7.5 Hz, Ar-H), 8.22 (1H, d, J 7.7 Hz, Ar-H); HRMS (ESI) Cald. for $C_{19}H_{27}O_{12}^+$ (M + H^+) 447.1497. Found 447.1483.

Benzoyl ß-D-maltoside **9**

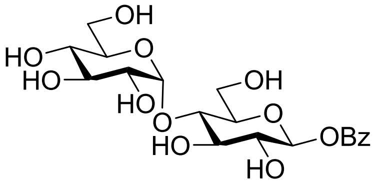

D-Maltose monohydrate (402 mg, 1.11 mmol), thiobenzoic acid (670 μL, 5.60 mmol) and triethylamine (1.6 mL, 11.4 mmol) were stirred in H_2O/MeCN (5:1, 4 mL), and the mixture was cooled to 0 °C. DMC **1** (580 g, 3.40 mmol) was then added portionwise and the mixture then allowed to stir for 30 mins. The reaction mixture was then diluted with H_2O (20 mL), washed with DCM (5 x 25 mL), and the aqueous phase was filtered through a 0.45 μm PTFE filter. Purification of the filtrate by semi-preparative RP-HPLC (column: Agilent 5 Prep-C18 (100 Å); eluent: A (water + 0.1 % trifluoroacetic acid) and B (MeCN + 0.1 % trifluoroacetic acid); sample was run at 25 mL/min with a gradient of: 0-30 min: 100% A, 30-40 min 100% B, 40-60 min: 100 % A, 60-70 min: 100% A; detection: 235 nm) afforded benzoyl ß-D-maltoside **9** (22.9 mg, 5 %) as a white solid, R_f 0.2 (1:4 MeOH:DCM); t_R = 4.82 mins; δ_H (400 MHz, D_2O)[6] 3.44 (1H, at, *J* 9.5 Hz, H-4), 3.61 (1H, dd, $J_{4a,5a}$ 10.5 Hz, $J_{5a,6a}$ 4.9 Hz, H-5a), 3.71-3.86 (8H, m, H-2a, H-3a, H-2b, H-3b, H-4b, H-5b, H-6b, H-6b'), 3.92-3.97 (2H, m, H-6a, H-6a'), 5.46 (1H, d, $J_{1b,2b}$ 3.9 Hz, H-1b), 5.84 (1H, d, $J_{1a,2a}$ 8.2 Hz, H-1a), 7.58 (2H, d, *J* 7.5 Hz, Ar-H), 7.74 (1H, at, *J* 7.5 Hz, Ar-H), 8.14 (2H, d, *J* 7.8 Hz, Ar-H); HRMS (ESI) Cald. for $C_{19}H_{25}O_{12}^{-iui}$ (M - H^+) 445.1351. Found 447.1339.

Benzoyl ß-D-maltotrioside **10**

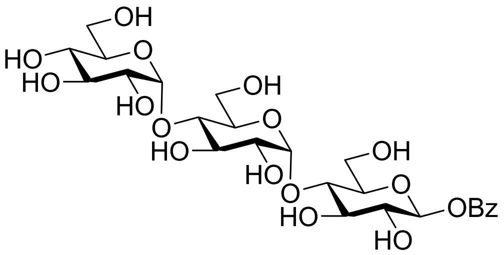

Maltotriose (600 mg, 1.20 mmol), thiobenzoic acid (700 μL, 5.90 mmol) and triethylamine (1.7 mL, 12.2 mmol) were stirred in H_2O/MeCN (5:1, 4 mL), and the mixture was cooled to 0 °C. DMC **1** (590 mg, 3.60 mmol) was then added portionwise and the mixture then allowed to stir for 30 mins. The reaction mixture was then diluted with H_2O (20 mL), washed with DCM

(5 x 25 mL), and the aqueous phase was filtered through a 0.45 μm PTFE filter. Purification of the filtrate by semi-preparative RP-HPLC (column: Agilent 5 Prep-C18 (100 Å); eluent: A (water + 0.1 % trifluoroacetic acid) and B (MeCN + 0.1 % trifluoroacetic acid); sample was run at 25 mL/min with a gradient of: 0-30 min: 100% A, 30-40 min 100% B, 40-60 min: 100 % A, 60-70 min: 100% A; detection: 235 nm) afforded benzoyl ß-D-maltotrioside **10** (20.7 mg, 3 %) as a white solid, R_f 0.1 (3:7 MeOH:DCM); t_R = 5.24 mins; δ_H (400 MHz, D_2O) 3.39 j(1H, m, H-5a), 3.54-3.98 (17H, m, H-2a, H-3a, H-4a, H-6a, H-6a', H-2b, H-3b, H-4b, H-5b, H-6b, H-6b', H-2c, H-3c, H-4c, H-5c, H-6c, H-6c'), 5.38 (1H, d, $J_{1c,2c}$ 4.0 Hz, H-1c), 5.40 (1H, d, $J_{1b,2b}$ 3.9 Hz, H-1b), 5.81 (1H, d, $J_{1a,2a}$ 8.2 Hz, H-1a), 7.55 (2H, d, *J* 7.5 Hz, Ar-H), 7.71 (1H, d, *J* 7.5 Hz, Ar-H), 8.10 (2H, d, *J* 7.8 Hz, Ar-H); δ_C (100 MHz, D_2O) 60.3 (t, C-6c), 60.5 (t, C-6b), 69.3 (d, C-5a), 71.2 (d, C-5c), 71.5 (d, C-3b), 71.7 (d, C-3c), 71.9 (d, C-5b), 72.7 (d, C-2b), 72.9 (d, C-2c), 73.3 (d, C-2a), 75.5 (d, C-4a), 75.9 (d, C-4c), 76.4 (d, C-3a), 76.9 (d, C-4b), 94.4 (d, C-1a), 99.5 (d, C-1b), 99.8 (d, C-1c), 128.1, 128.8, 129.9, 134.6 (Ar-C), 166.9 (s, $\underline{C}OC_6H_5$); HRMS (ESI) Cald. for $C_{25}H_{35}O_{17}^-$ (M - H^+) 607.1880. Found 607.1874.

Spectra Data

Benzoyl 2-Acetamido-2-deoxy-D-glucopyranoside **3**

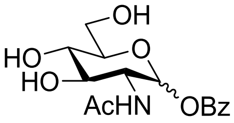

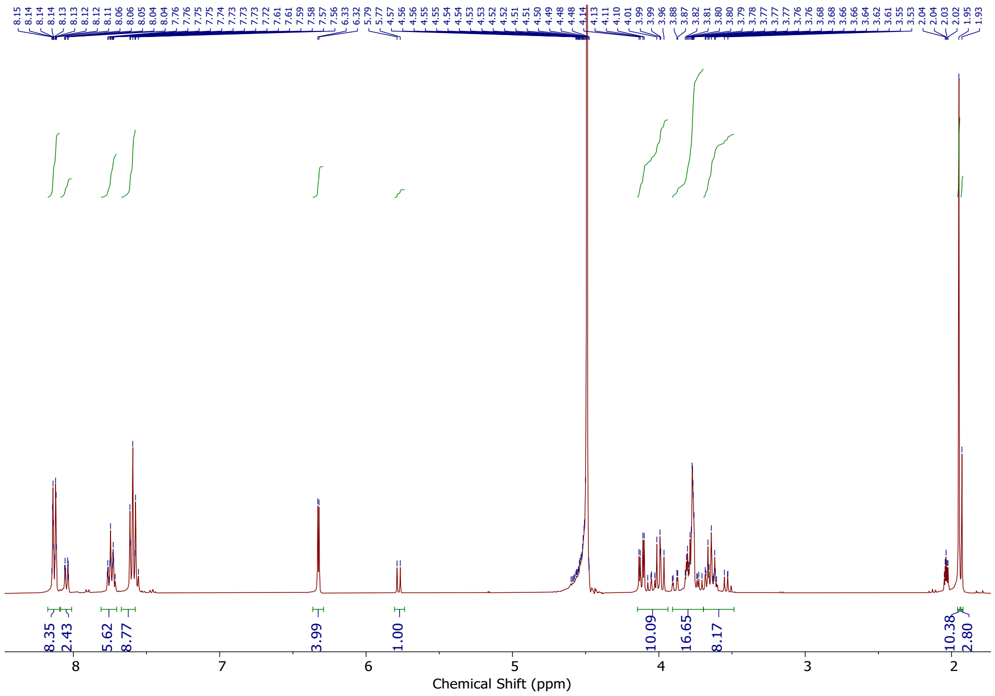

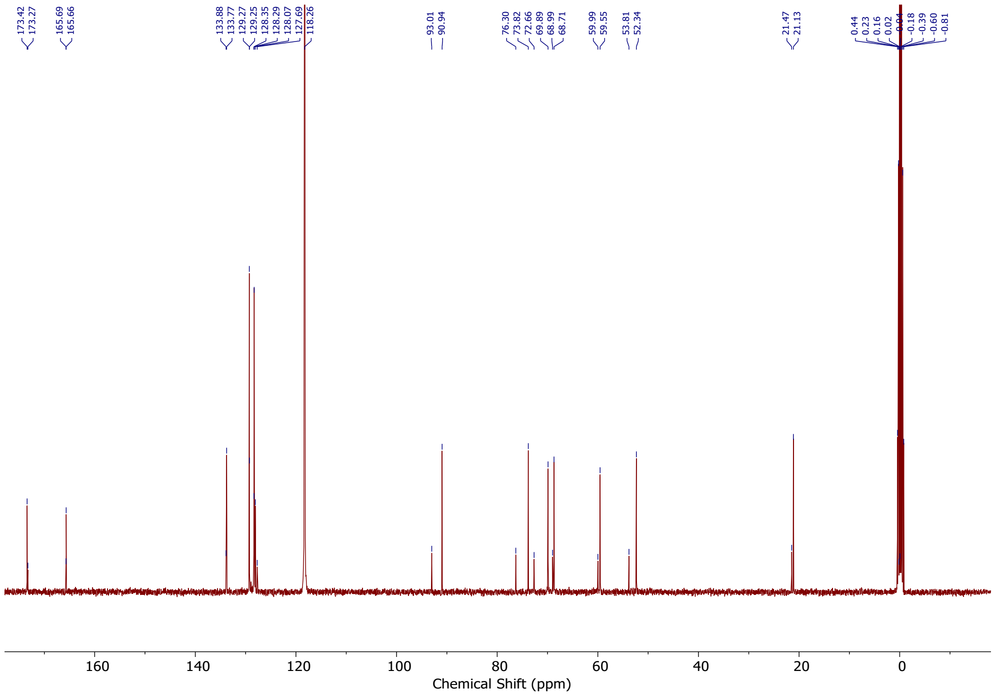

Benzoyl ß-D-glucopyranoside **5**

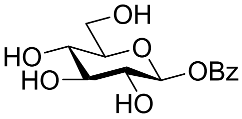

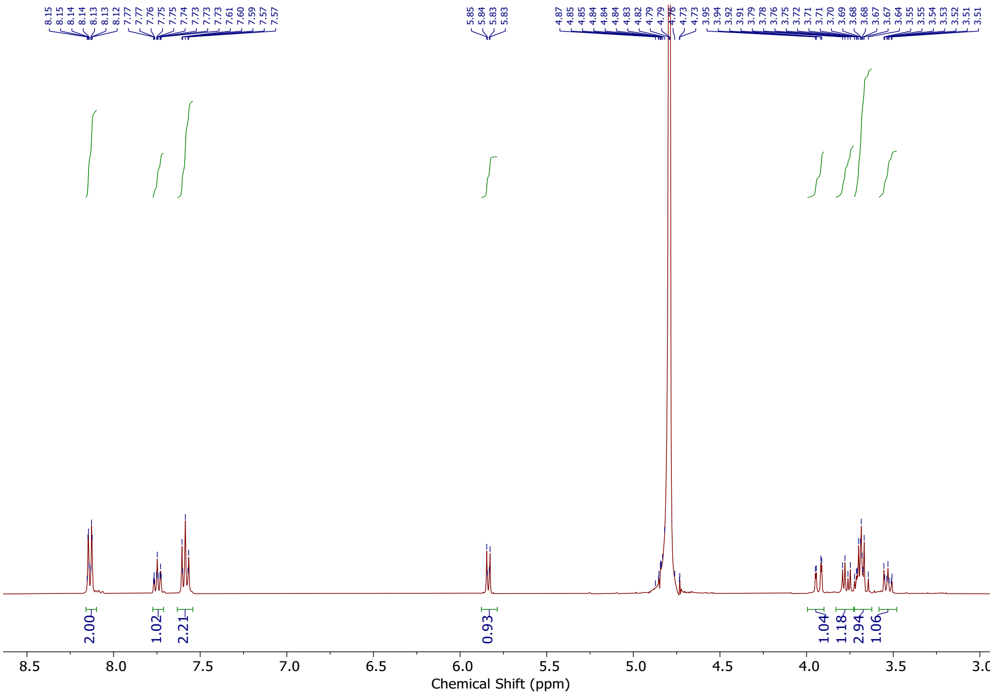

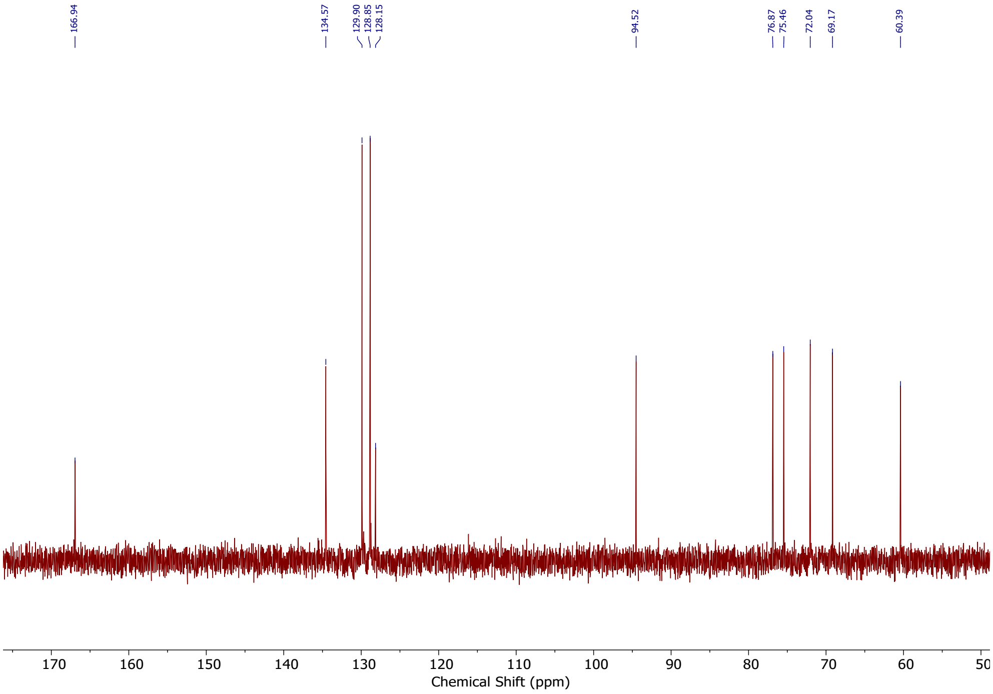

Benzoyl α-D-mannopyranoside **7**

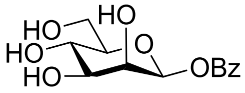

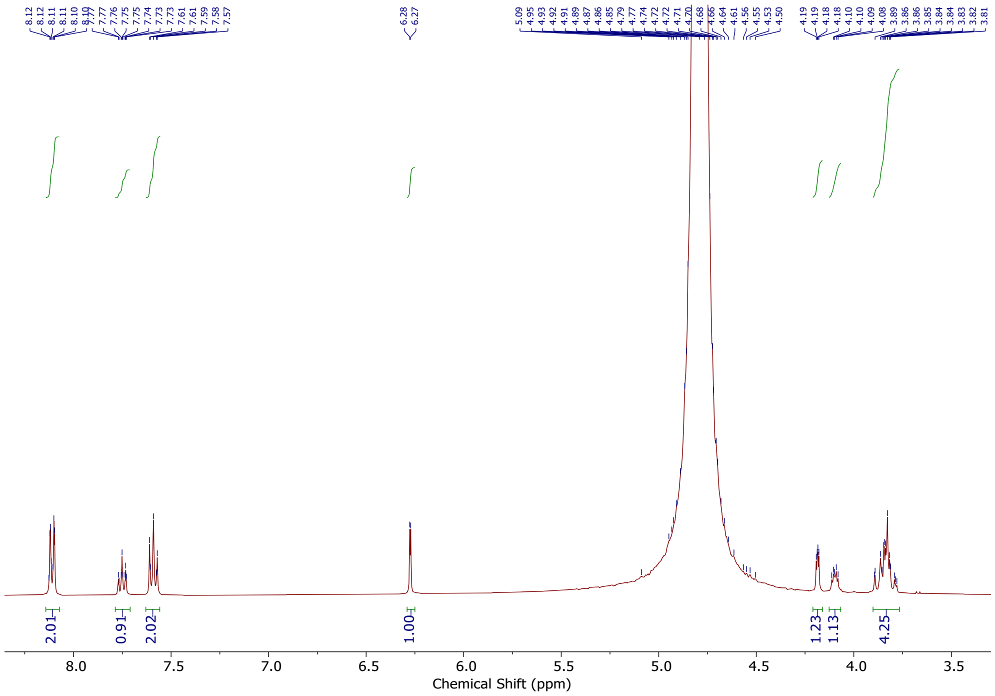

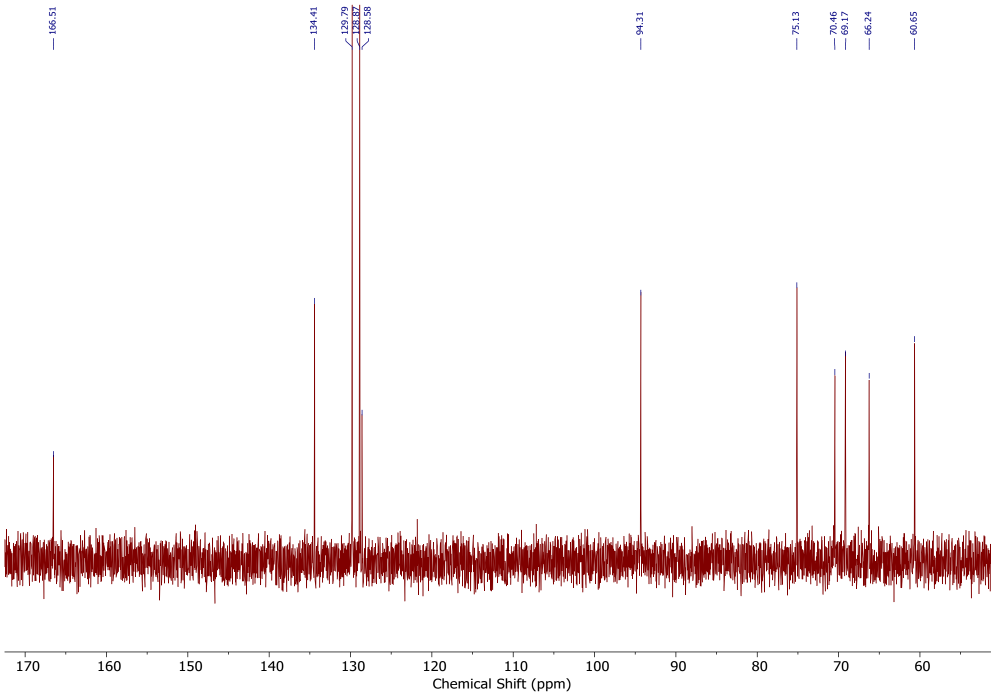

Benzoyl ß-D-lactoside **8**

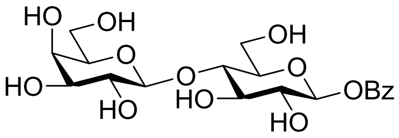

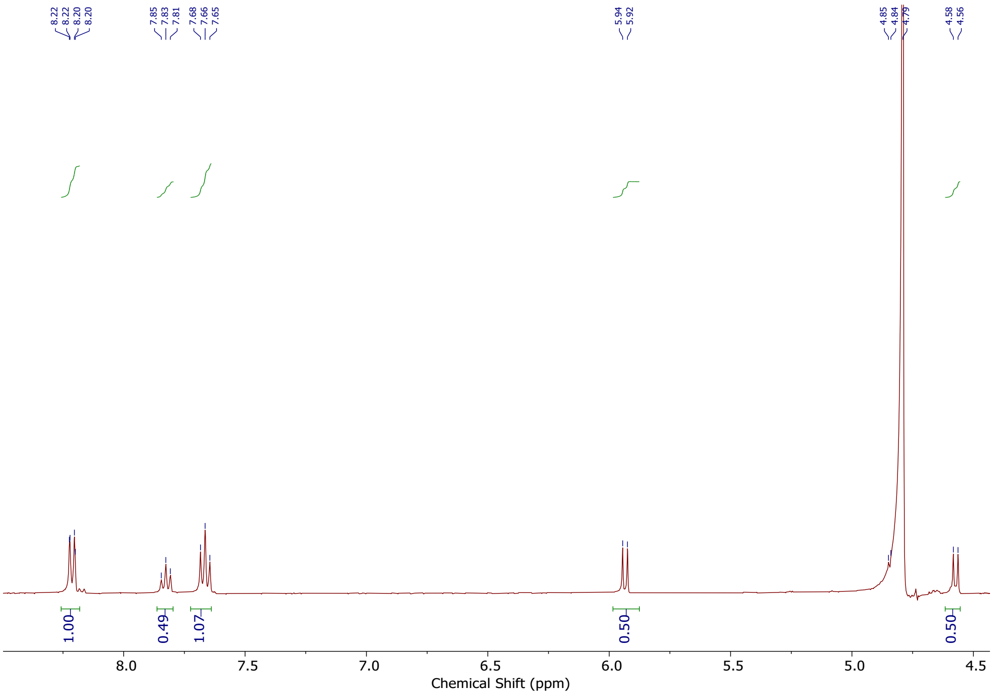

Benzoyl ß-D-maltoside **9**

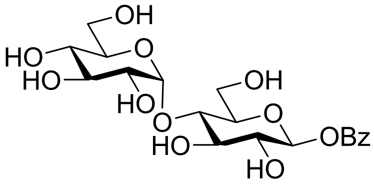

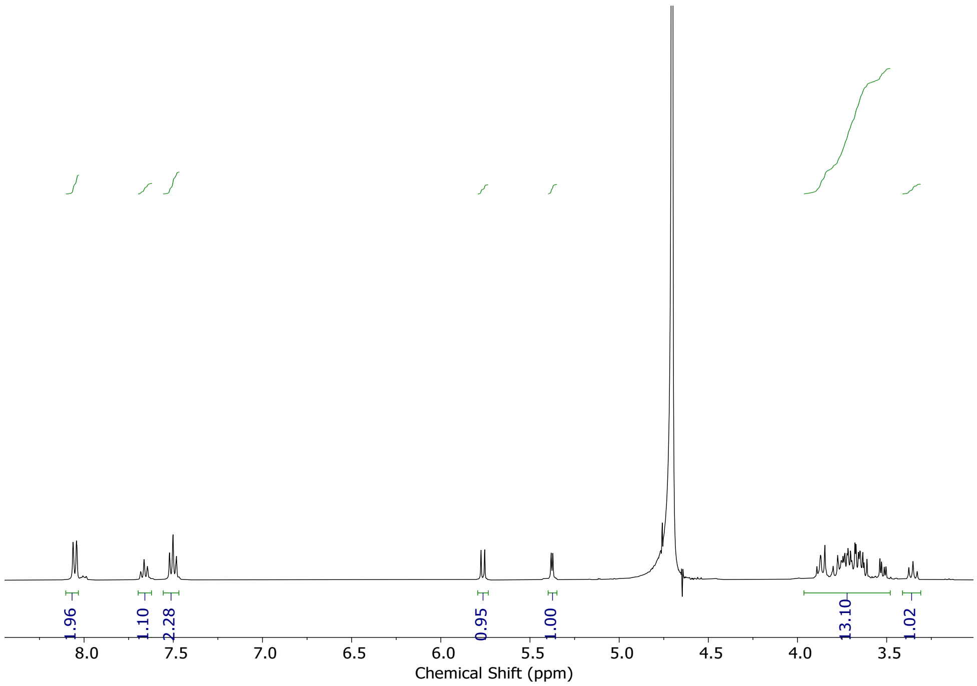

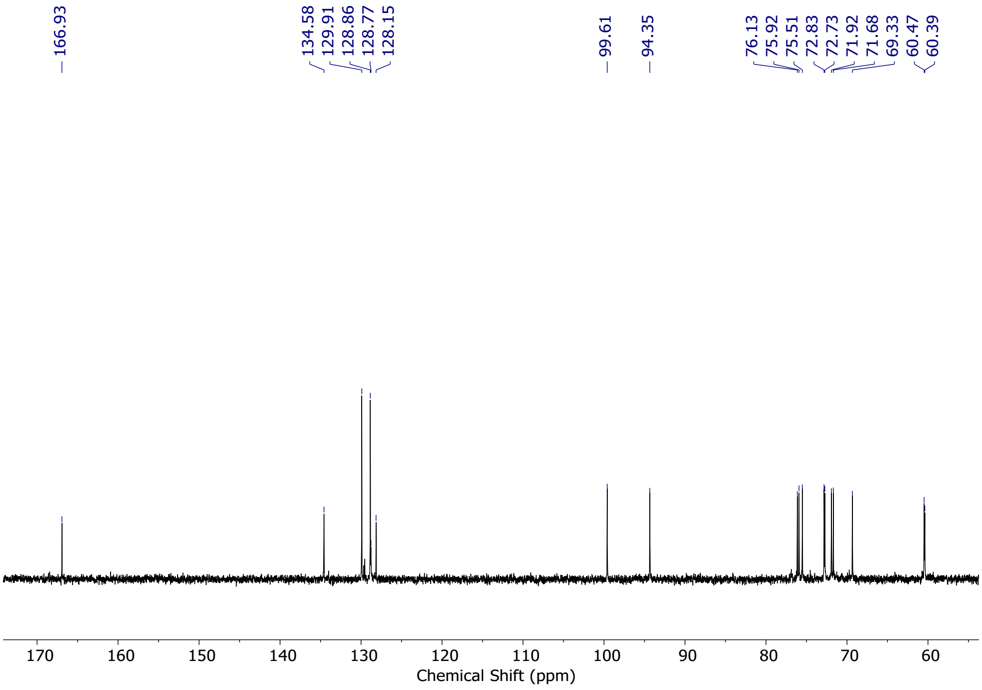

Benzoyl ß-D-maltotrioside **10**

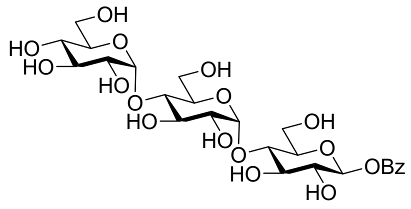

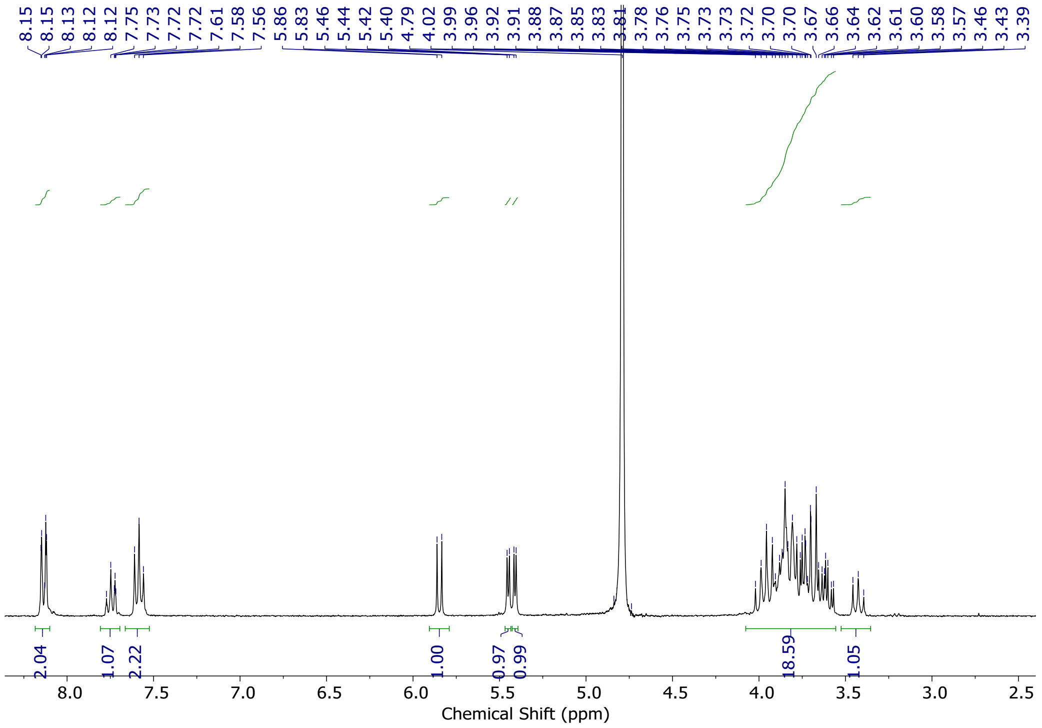

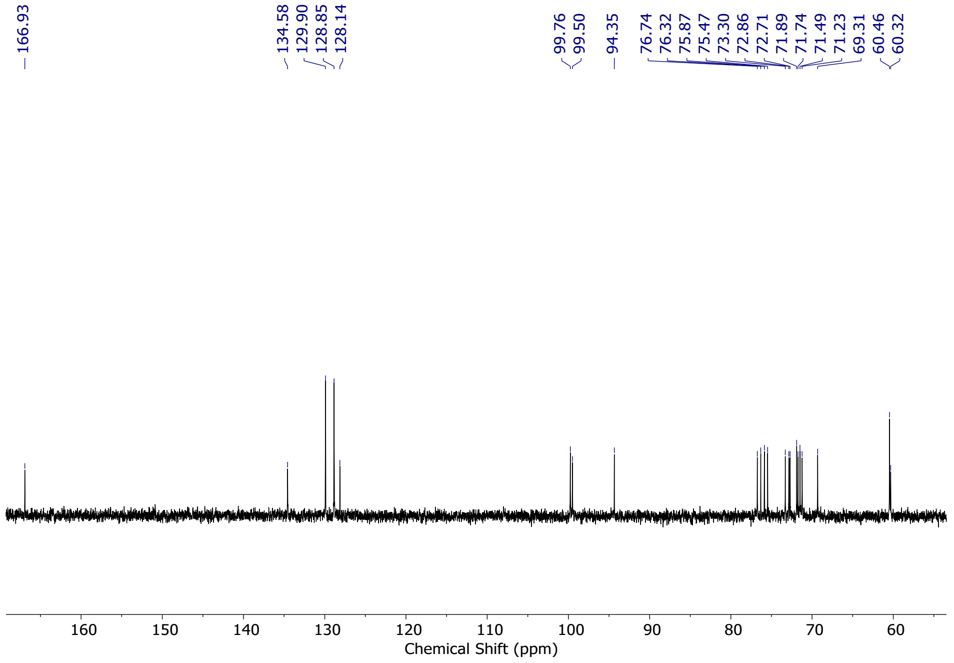

References

1. Pillet, L.; Lim, D.; Almulhim, N.; Benitez-Mateos, A. I.; Paradisi, F., *Chem Commun.* **2022,** *58* (86), 12118-12121.
2. Bock, K.; Lundt, I.; Pedersen, C., *Tetrahedron Lett.* **1973,** *14* (13), 1037-1040.
3. Almulhim, N.; Moody, N. R.; Paradisi, F., *Appl Microbiol Biotechnol* **2020,** *104* (10), 4407-4415.
4. Wilkins, M. R.; Gasteiger, E.; Bairoch, A.; Sanchez, J. C.; Williams, K. L.; Appel, R. D.; Hochstrasser, D. F., *Methods Mol Biol* **1999,** *112*, 531-52.
5. Lim, D.; Brimble, M. A.; Kowalczyk, R.; Watson, A. J.; Fairbanks, A. J., *Angew Chem Int Ed* **2014,** *53* (44), 11907-11.
6. Dey, K.; Jayaraman, N.,*Chem Commun* **2022,** *58* (13), 2224-2227.
